# Antibiotic Use at the End of Life: Current Practice and Ways to Optimize

**DOI:** 10.1177/10499091241266986

**Published:** 2024-07-19

**Authors:** Minji Kang, Winnie S. Wang, Zieanna Chang

**Affiliations:** 1 Division of Infectious Diseases and Geographic Medicine, Department of Internal Medicine, 12334 UT Southwestern Medical Center, Dallas, TX, USA; 2 Division of General Internal Medicine, Department of Internal Medicine, 12334 UT Southwestern Medical Center, Dallas, TX, USA

**Keywords:** antibiotic stewardship, end of life care, palliative care

## Abstract

Infections are common complications in end of life (EOL). However, clinicians have minimal guidance regarding antibiotic decision-making in EOL care, leading to the overuse of antibiotics. While symptom relief is frequently cited as a major reason for antibiotic use in EOL, antibiotics have not been shown to provide significant improvement in symptoms outside of urinary tract infections. In addition, when prognosis is expected to be in the range of days to weeks, antibiotics have not been shown to provide significant survival benefit. Antibiotics can be beneficial in EOL care in appropriate scenarios, but the current widespread use of antibiotics in EOL requires reevaluation. There needs to be broader efforts to think about antibiotics like other invasive medical procedures in which benefits and risks are weighed, recognizing that not all patients in EOL who receive antibiotics will benefit. In addition, during care planning process, discussing and documenting antibiotic preferences will be beneficial. Non-antibiotic symptom management should be incorporated to plan of care when infection is suspected. Ultimately, the use of antibiotics at EOL should be for the clear benefit for the recipient and should be guided by the type of infection and its clinical course, patients’ primary disease and its prognosis, and the preferences of patients or surrogate decision makers.

## Introduction

Patients approaching the end of life (EOL) are susceptible to infections given their debility, comorbidities, and other factors like the presence of invasive devices and frequent exposure to healthcare settings.^1^ Infections increase the symptom burden of patients approaching EOL, but infections can also be part of the dying process and may often be the final cause of death. The decision to initiate, withhold, or withdraw antibiotics at the EOL is controversial and multifaceted. Even though infections are common complications in EOL that lead to hospital admissions, clinicians have minimal guidance regarding antibiotic decision-making.

## Clinicians’ Perception of Antibiotics in End of Life

Physicians may overestimate the benefits of antibiotics at EOL while underestimating their risks. In a survey characterizing physician perception of antibiotic use at EOL, while 96% agreed that “the overuse of antibiotics contributes to antibiotic resistance,” only 50% agreed that “antimicrobial use in end of life care contributes to antibiotic resistance.” Physicians may overestimate the efficacy of antibiotics at EOL with more than 70% of physicians agreeing that infections can be cured or that the progression of infection can be prevented with antibiotics at EOL. Physicians emphasize respecting patient autonomy and a shared decision-making model in EOL care with 86% of physicians agreeing that it is important to respect the patient’s request to continue antibiotic treatment. In addition, physicians found alleviation of pain and work of breathing to be important reasons to continue antibiotics at EOL.^2^

## Antibiotic Use in End of Life

Such perception of antibiotics is reflected in the prevalence of antibiotic use at EOL. While there is wide variability in the patient population, study setting, definition of EOL, and definition of infections in these studies, the overuse of antibiotics during inpatient hospitalizations is well documented in EOL care especially among patients with advanced cancer and dementia.

Many retrospective cohort and prospective studies have demonstrated the wide use of antibiotics in patients with advanced cancer approaching EOL.^3-10^ In the inpatient hospital setting, antibiotic use is frequent and seen in 46% to 87% of patients during the last week of life.^3,5,11,12^ Even after transitioning to comfort measures, patients continued to receive antibiotics with approximately 40% receiving antibiotics until the date of death or discharge.^4,9,12^ Antibiotic use was associated with 34% longer length of stays.^9^ Respiratory tract and urinary tract infections were most common, followed by skin and soft tissue infections.^6,10^ Antibiotic use was less frequently seen in outpatient hospice settings, though still prevalent.^7,13,14^ For instance, in a retrospective cohort study, 58% received antibiotics in the last week of life in acute care settings in comparison to 22% in outpatient hospice settings.^7^

Similarly, high utilization of antibiotics in patients with advanced dementia who are approaching EOL is also well documented in the literature. Antibiotic use in patients with advanced dementia is highly prevalent in inpatient hospital settings.^15,16^ The proportion of patients receiving antibiotics, the number of antibiotic therapy courses, and days of therapy were all found to increase as patients approached death.^17^ The absence of a do-not-hospitalize order, a primary language other than English, and unstable vital signs were independently associated with more invasive therapy.^18^ Male sex was associated with 33% higher odds of receiving antibiotics in the last 30 days of life.^19^ Similar to patients with advanced cancer, respiratory tract and urinary tract infections were most common, followed by skin and soft tissue infections.^17,20^ Quinolones were the most frequently prescribed agents.^17,20^ Antibiotic use was less common in patients enrolled in hospice with 27% of patients receiving antibiotics during the last 7 days of life in a nationwide analysis of antibiotic use in hospice care.^21^ Among patients enrolled in hospice, patients who received antibiotics were younger, and had longer durations of hospice enrollment.^21^

Regardless of the diagnosis, antibiotics are frequently continued even after transitioning to comfort care as seen in 41 to 77% of patients.^22,23^ Patients in intensive care units were less likely to receive antibiotics than those on medicine services.^22,23^ Similarly, when discharging to hospice from acute care settings, antibiotics were prescribed in 18 to 33% of individuals.^24-26^ This may be because discharge medications were addressed only in 62% of patients and orders for antibiotic use were available in Physicians Orders for Life Sustaining Treatment (POLST) form in only 48% of participants. When orders for antibiotic use were documented in POLST, 47% of forms documented orders to use antibiotics if medically indicated, and 43% documented to determine use and limitation of antibiotics at the time when infection occurred.^26^

## Impact Subspecialty Consultation on Antibiotic Use

Prior studies have reported increased deprescribing with palliative care consultation.^27^ For instance, the percentage of potentially inappropriate medications decreased significantly from 21% to 14% after palliative care consultation.^27^ Similarly, among patients with terminal cancer at EOL, patients with palliative care consultation had significantly lower antibiotic use than patients without palliative care consultation.^28^

On the other hand, infectious diseases consultations were associated with increased antibiotic days of therapy. Infectious diseases consultation rate was lower for patients who are at EOL with advanced directives compared to those without advanced directives. Among patients with advanced directives, mean antibiotic days of therapy were higher among patients with infectious diseases consultations than those who did not have infectious diseases consultation.^29^ However, the results need to be interpreted with caution as patients who had infectious diseases consultation likely had more complicated or severe infections that necessitated the consultation.

While infectious diseases physicians are the leaders of antibiotic stewardship programs (ASPs), they may not necessarily be equipped to determine if antibiotics are concordant with patients’ goals of care, or if it is indicated based on the patients’ prognosis. This finding emphasizes the need for palliative care consultation to facilitate goals of care discussions with patients, family, and various multidisciplinary teams. Palliative care consultations can help clarify patients’ values and preferences to aid infectious diseases physicians as well as other care teams to determine the appropriateness of antibiotics and other therapy.

## Benefits of Antibiotics at End of Life

When physicians were asked about reasons for initiating antibiotics, 100% noted that it was to relieve symptoms.^30^ However, while symptom relief is most frequently cited as a major reason for antibiotic use at EOL, antibiotics may not necessarily provide symptom relief outside of urinary tract infections. There have been no randomized clinical trials evaluating the efficacy of antibiotics in symptom relief with nearly all studies retrospective in nature. In patients with terminal cancer, symptom relief with antibiotics was only seen in 15 to 48%.^8,11,14,28,30^ Antibiotics appear to provide symptom relief in urinary tract infections (UTI) with up to 79 percent of patients with terminal cancer experiencing resolution or improvement of dysuria.^13,14,30^ However, minimal symptom improvement was seen in respiratory tract, mouth/pharynx, or skin/subcutaneous infections.^13,14,30^ In patients started on antibiotic treatment to avoid or treat sepsis, symptom relief was only seen in 50 percent.^11^ Other symptoms such as cough, dyspnea, pain, or confusion did not show statistical improvement despite antibiotics.^30^ After initiation of antibiotics, fever was controlled only in 48%.^28^ Similar results have been shown in patients with end stage dementia. Due to aspiration, pneumonia is a frequent complication of advanced dementia. When comparing patients who received antibiotics for pneumonia to those who did not, patients receiving any form of antimicrobial treatment for pneumonia had lower scores for Symptom Management at End-of-Life in Dementia Scale compared to those who were untreated.^31^ In addition, fever was frequently seen in patients with advanced dementia and frequency of fever increased with progression of dementia. Antibiotic treatment did not impact the outcome of fevers and fevers frequently resolved regardless of antibiotic use.^32^

In addition to symptom relief, another reason to initiate antibiotics was to prolong survival. Data regarding this, however, appear mixed. Patients’ survival has not been shown to be affected by the presence of infection or the use of antibiotics.^13,14^ In another prospective study of residents with advanced dementia at US Department of Veterans Affairs nursing homes, antibiotics were associated with reduced 10 day mortality, but not with 1 month or 6 months mortality.^33^ In another study, however, when comparing patients who received antibiotic treatment for pneumonia to patients who did not, antibiotics for pneumonia had a 273 day survival benefit compared to no treatment for pneumonia.^31^ Overall, when prognosis is predicted to be short in the range of days to weeks or weeks to months, antibiotics are unlikely to provide significant survival benefit and may prolong life by a few short days at most.^33^ However, for patients whose prognosis is longer in the range of months to years, antibiotics may offer survival benefit.

## Risks of Antibiotics at End of Life

The limitations of antibiotics are further compounded by the fact that antibiotics in EOL are initiated due to clinical suspicion for infection despite lack of clear evidence of infection. For instance, among patients with advanced dementia in nursing homes, only 16% of episodes in which antibiotics were initiated for presumed UTI met minimal criteria to initiate antibiotics based on signs and symptoms of UTI.^34^ Similarly, in a retrospective study aimed at determining the percentage of antibiotics that were appropriately initiated based on Loeb’s Minimum Criteria (LMC) for Antibiotic Initiation Tool, only 42% of antibiotics were noted to be appropriate.^35^ In another study, among patients who received antibiotics, 15% had documentation for potential indication.^21^

On an individual level, overuse of antibiotics can lead to adverse effects, increased length of stay, and increased cost.^36^ Adverse effects due to antibiotics include allergic reactions, side effects like gastrointestinal issues, drug-drug interactions, and *Clostrioides difficile* infection.^37^ Evaluation for infection and treatment with antibiotics can potentially be invasive and cause burden in terminally ill patients. In addition to the impact that antibiotics have on an individual level, there are concerns from a broader public health perspective that antibiotic overuse can lead to emergence and spread of multidrug resistant organisms (MDROs). More than 30% of patients who are at EOL were noted to have MDROs with 10% having more than 1 MDRO.^29^ In addition, in the ICU, EOL treatment as well as the absence of DNR order was associated with the acquisition of MDROs.^38^ This pattern has been shown to be true in outpatient settings as well. MDRO colonization was seen in 67% of nursing home residents with advanced dementia at some point over a 12-month period. Greater exposure to fluoroquinolones and third and fourth generation cephalosporins were significantly associated with MDRO acquisition.^20^

## Antibiotic Stewardship Program at End of Life

The current guideline for the implementation of ASPs by Infectious Diseases Society of America (IDSA) and Society for Healthcare Epidemiology of America (SHEA) recommend that ASPs provide support in decisions regarding antimicrobial use in terminally ill patients.^39^ Despite this, only a minority of ASPs are involved in decisions regarding antibiotic prescribing in patients receiving comfort care. In a survey of ASPs in various acute care hospitals, 64% of ASPs monitored antibiotic use for end-of-life care and 36% of ASPs provided guidance for patients receiving comfort care only. Only 14% of surveyed hospitals noted integrating antimicrobials into institutional EOL care guidelines.^40^

## Ethics of Antibiotic Use at End of Life

The ethics of withdrawing or withholding life sustaining treatment at EOL has been deliberated extensively. It has been found to be acceptable when the informed patient or surrogate decision maker (SDM) determines that the benefit of life sustaining treatment no longer outweighs the risk.^41^ Antibiotics are one of these therapies. However, clinicians commonly experience doubt when counseling patients and families regarding antibiotics as the risks of interventions like CPR, intubation, surgery, or chemotherapy may seem more evident than those of antibiotics, anticoagulation, or micronutrient supplements.

The ethical principle of autonomy holds that patients have the right to make decisions that align with their values. For patients to make an informed decision, clinicians must provide a summary of risks, benefits, and alternatives while accounting for the components of care that patients value the most. For example, a patient whose primary goal is to return home can make a better decision when informed that intravenous antibiotic is preventing his or her discharge. The patient may be counseled that further treatment may not significantly extend his or her life or improve pain from an incurable abscess. This counseling varies widely from patient to patient. Another patient may hope to spend more time in the hospital out of concern for the surviving family’s psychological well-being. For such patients, information about which therapies cannot be replicated at home is less useful. By exploring what is most important to the patient, clinicians can better tailor their discussions of risks and benefits.

Another challenge faced is the perception that families are requesting non-beneficial treatments despite clinicians’ assessments or recommendations.^42^ In many, but not all scenarios, questions or statements about risks or benefits reflect a mixture of emotions and cognitive inquiry. Family members may ask, “wouldn’t stopping antibiotics shorten his life?” This can engender concerns that family members may be unwilling to accept the patient’s terminal state. However, skillful clinicians may recognize that this question may be a request for reassurance that the surrogate would not be “responsible” for the patient’s death by accepting a recommendation to discontinue antibiotics. Surrogates may also request more information about whether and why antibiotics are no longer beneficial. Moving through the emotional response creates space to engage in an objective clinical deliberation, thus maintaining autonomy while respecting the other ethical principles of beneficence, non-maleficence, and justice.

## Improving Antibiotic Use at End of Life

It is evident that the current use of antibiotics in EOL requires reevaluation. Currently, clinicians have minimal guidance regarding antibiotic decision-making at EOL. Risks of interventions like CPR, intubation, surgery, or chemotherapy may seem more evident than those of antibiotics. However, there needs to be broader efforts to think about antibiotics like other invasive medical procedures in which benefits and risks are weighed, recognizing that not all patients in EOL who receive antibiotics will benefit. The use of antibiotics at EOL should be for the clear benefit for the patient and should be guided by the type of infection and its clinical course, the primary disease and its prognosis, and the preferences of patients or SDMs.

When an advanced life-limiting illness is identified, patient and SDMs should be counseled on common infectious complications and their clinical course. Setting realistic expectation with patients and SDMs that infections are common at EOL and that not all infections are reversible acute complications but are a natural part of the dying process is crucial. For instance, in patients with advanced dementia, SDMs should be made aware that aspiration pneumonia can occur; in patients with cholangiocarcinoma, patients should be counseled that recurrent cholangitis can occur due to malignant obstruction. With expectations discussed, incorporating antibiotic preferences as part of the advanced care planning process can help optimize its use. Overall, antibiotics are infrequently discussed during advanced care planning conversation. Only 33% of SDMs noted that they were counseled by clinicians that infections were common in advanced dementia. Only 38% were counseled about antibiotic use and only 45% were asked about preferences regarding antibiotic use.^20^ However, when antibiotics are discussed, most patients and SDMs preferred no antibiotic or symptomatic use only. For instance, when antibiotics were discussed with 255 patients with advanced cancer entering a community-based hospice program, 79% chose either no antibiotics or symptomatic use only.^14^ Patients specifying limited antibiotics on Physician Orders for Life Sustaining Treatment (POLST) form were noted to have significantly lower antibiotic days of therapy.^43^ Antibiotic use at EOL should become a routine part of advanced care planning conversation and benefits and potential adverse effects of antibiotics should be discussed along with patient or surrogate decision makers preferences.

When concerns for infection arise, it is important to elucidate what the current symptoms are and if the symptoms are causing discomfort to the patient to ascertain if infection is truly present. Infections can be difficult to diagnose with antibiotics in EOL frequently initiated due to clinical suspicion of infection despite the lack of clear evidence of infection.^34,35^ If preferences for antibiotics and goals of care have not been addressed in advance care planning, further discussion on what the evaluation of suspected infection entails and the risks and benefits of evaluating and treating an infection should be discussed. For some patients, they may desire all life prolonging measures, which may include hospitalization, procedures, and intravenous antibiotics to treat the infection. For other patients, they may desire comfort measures only. It is important to identify achievable goals in patients, taking into account what type of infection may be present and what their underlying disease is. For instance, for urinary tract infections, antibiotics can be helpful for symptomatic improvement even in patients who may desire comfort measures only. However, in case of a pelvic abscess in setting of unresectable rectal cancer, it is important to note that source control is not feasible and that antibiotics are likely to only provide temporary relief. In addition, given limitations of antibiotics for symptom management for certain infectious complications, non-antibiotic symptom management also should be incorporated to plan of care. In a retrospective study of patients with dementia who were in their last month of life, 72% received antibiotics, while 37% received anxiolytics and 8% received antidepressants.^44^ Another study showed that morphine was more frequently provided when antibiotics were withheld.^33^ While antibiotics are appropriate for symptom management in urinary tract infections, it may be of little benefit in infections like pneumonia where antipyretics and opioids for dyspnea may be more beneficial than antibiotics.

Finally, in general, further collaboration between ASPs and palliative care practitioners is necessary. Given the magnitude of antibiotic use during EOL care, this topic is a fundamental aspect of ASPs. ASPs in conjunction with palliative care practitioners can develop clear guidelines to help with decision-making about antibiotics for in-hospital palliative care and transitions to hospice. There is a need for regulatory bodies to develop best practice guideline pertaining to antibiotic use at end of life. Guidelines should focus on when infection should be suspected at EOL, standard of care for ethically appropriate antibiotic use, and alternative treatment options for symptom management at EOL.

## Conclusion

The decision to initiate, withhold, or withdraw antibiotics at EOL is multifaceted. Even though infections are common complications in EOL, healthcare providers have minimal guidance regarding antibiotic decision-making. Antibiotics can be beneficial in EOL care in appropriate scenarios, but the current widespread use of antibiotics in EOL requires reevaluation. The use of antibiotics at EOL should be for the clear benefit for the individual who receives them and should be guided by the type of infection and its clinical course, primary disease and its prognosis, and the preferences of patients or surrogate decision makers.

## References

[1] Vitetta L Kenner D Sali A . Bacterial infections in terminally ill hospice patients. J Pain Symptom Manag. 2000;20(5):326-334. doi:10.1016/s0885-3924(00)00189-5. 11068154

[2] Gaw CE Hamilton KW Gerber JS Szymczak JE . Physician perceptions regarding antimicrobial use in end-of-life care. Infect Control Hosp Epidemiol. 2018;39(4):383-390. doi:10.1017/ice.2018.6. 29428002

[3] Abduh Al-Shaqi M Alami AH Zahrani AS Al-Marshad B Muammar AB Al-Shahri MZ . The pattern of antimicrobial use for palliative care in-patients during the last week of life. Am J Hosp Palliat Care. 2012;29(1):60-63. doi:10.1177/1049909111406900. 21676985

[4] Azad AA Siow SF Tafreshi A Moran J Franco M . Discharge patterns, survival outcomes, and changes in clinical management of hospitalized adult patients with cancer with a do-not-resuscitate order. J Palliat Med. 2014;17(7):776-781. doi:10.1089/jpm.2013.0554. 24865307

[5] Baek SK Chang HJ Byun JM Han JJ Heo DS . The association between end-of-life care and the time interval between provision of a do-not-resuscitate consent and death in cancer patients in Korea. Cancer Res Treat. 2017;49(2):502-508. doi:10.4143/crt.2016.073. 27586675 PMC5398395

[6] Pereira J Watanabe S Wolch G . A retrospective review of the frequency of infections and patterns of antibiotic utilization on a palliative care unit. J Pain Symptom Manag. 1998;16(6):374-381. doi:10.1016/s0885-3924(98)00093-1. 9879162

[7] Oneschuk D Fainsinger R Demoissac D . Antibiotic use in the last week of life in three different palliative care settings. J Palliat Care. 2002;18(1):25-28. 12001399

[8] Oh DY Kim JH Kim DW , et al. Antibiotic use during the last days of life in cancer patients. Eur J Cancer Care. 2006;15(1):74-79. doi:10.1111/j.1365-2354.2005.00603.x. 16441680

[9] Datta R Zhu M Han L Allore H Quagliarello V Juthani-Mehta M . Increased length of stay associated with antibiotic use in older adults with advanced cancer transitioned to comfort measures. Am J Hosp Palliat Care. 2020;37(1):27-33. doi:10.1177/1049909119855617. 31185722 PMC6868290

[10] Lam PT Chan KS Tse CY Leung MW . Retrospective analysis of antibiotic use and survival in advanced cancer patients with infections. J Pain Symptom Manag. 2005;30(6):536-543. doi:10.1016/j.jpainsymman.2005.06.005. 16376740

[11] Helde-Frankling M Bergqvist J Bergman P Björkhem-Bergman L . Antibiotic treatment in end-of-life cancer patients-a retrospective observational study at a palliative care center in Sweden. Cancers. 2016;8(9):84. doi:10.3390/cancers8090084. 27608043 PMC5040986

[12] Thompson AJ Silveira MJ Vitale CA Malani PN . Antimicrobial use at the end of life among hospitalized patients with advanced cancer. Am J Hosp Palliat Care. 2012;29(8):599-603. doi:10.1177/1049909111432625. 22218916

[13] Reinbolt RE Shenk AM White PH Navari RM . Symptomatic treatment of infections in patients with advanced cancer receiving hospice care. J Pain Symptom Manag. 2005;30(2):175-182. doi:10.1016/j.jpainsymman.2005.03.006. 16125033

[14] White PH Kuhlenschmidt HL Vancura BG Navari RM . Antimicrobial use in patients with advanced cancer receiving hospice care. J Pain Symptom Manag. 2003;25(5):438-443. doi:10.1016/s0885-3924(03)00040-x. 12727041

[15] NourhashémiF GilletteS CantetC , et al. End-of-life care for persons with advanced alzheimer disease: design and baseline data from the ALFINE study. J Nutr Health Aging. 2012;16(5):457-461. doi:10.1007/s12603-011-0333-9.22555791

[16] Ahronheim JC Morrison RS Baskin SA Morris J Meier DE . Treatment of the dying in the acute care hospital. Advanced dementia and metastatic cancer. Arch Intern Med. 1996;156(18):2094-2100. 8862102

[17] D’Agata E Mitchell SL . Patterns of antimicrobial use among nursing home residents with advanced dementia. Arch Intern Med. 2008;168(4):357-362. doi:10.1001/archinternmed.2007.104. 18299489 PMC2670184

[18] Chen JH Lamberg JL Chen YC , et al. Occurrence and treatment of suspected pneumonia in long-term care residents dying with advanced dementia. J Am Geriatr Soc. 2006;54(2):290-295. doi:10.1111/j.1532-5415.2005.00524.x. 16460381

[19] Stall NM Fischer HD Fung K , et al. Sex-specific differences in end-of-life burdensome interventions and antibiotic therapy in nursing home residents with advanced dementia. JAMA Netw Open. 2019;2(8):e199557. doi:10.1001/jamanetworkopen.2019.9557. 31418809 PMC6704739

[20] Mitchell SL Shaffer ML Loeb MB , et al. Infection management and multidrug-resistant organisms in nursing home residents with advanced dementia. JAMA Intern Med. 2014;174(10):1660-1667. doi:10.1001/jamainternmed.2014.3918. 25133863 PMC4188742

[21] Albrecht JS McGregor JC Fromme EK Bearden DT Furuno JP . A nationwide analysis of antibiotic use in hospice care in the final week of life. J Pain Symptom Manag. 2013;46(4):483-490. doi:10.1016/j.jpainsymman.2012.09.010. PMC3723720 23317761

[22] Fins JJ Miller FG Acres CA Bacchetta MD Huzzard LL Rapkin BD . End-of-life decision-making in the hospital: current practice and future prospects. J Pain Symptom Manag. 1999;17(1):6-15. doi:10.1016/s0885-3924(98)00109-2. 9919861

[23] Merel SE Meier CA McKinney CM Pottinger PS . Antimicrobial use in patients on a comfort care protocol: a retrospective cohort study. J Palliat Med. 2016;19(11):1210-1214. doi:10.1089/jpm.2016.0094. 27309999 PMC5105343

[24] Furuno JP Noble BN Horne KN , et al. Frequency of outpatient antibiotic prescription on discharge to hospice care. Antimicrob Agents Chemother. 2014;58(9):5473-5477. doi:10.1128/AAC.02873-14. 25001299 PMC4135830

[25] Kadoyama KL Noble BN Izumi S , et al. Frequency and documentation of medication decisions on discharge from the hospital to hospice care. J Am Geriatr Soc. 2019;67(6):1258-1262. doi:10.1111/jgs.15860. 30854629

[26] Servid SA Noble BN Fromme EK Furuno JP . Clinical intentions of antibiotics prescribed upon discharge to hospice care. J Am Geriatr Soc. 2018;66(3):565-569. doi:10.1111/jgs.15246. 29345756 PMC5849491

[27] Marin H Mayo P Thai V , et al. The impact of palliative care consults on deprescribing in palliative cancer patients. Support Care Cancer. 2020;28(9):4107-4113. doi:10.1007/s00520-019-05234-w. 31867703

[28] Kim JH Yoo SH Keam B Heo DS . The impact of palliative care consultation on reducing antibiotic overuse in hospitalized patients with terminal cancer at the end of life: a propensity score-weighting study. J Antimicrob Chemother. 2022;78(1):302-308. doi:10.1093/jac/dkac405. 36424671

[29] Fedorowsky R Bachner YG Borer A Ciobotaro P Kushnir T . Use of antibiotics among end-of-life hospitalized patients with advanced directives: status examination and association with infectious disease consultation and physician burnout. Infect Control Hosp Epidemiol. 2019;40(11):1222-1228. doi:10.1017/ice.2019.203. 31455445

[30] Mirhosseini M Oneschuk D Hunter B Hanson J Quan H Amigo P . The role of antibiotics in the management of infection-related symptoms in advanced cancer patients. J Palliat Care. 2006;22(2):69-74. 17265658

[31] Givens JL Jones RN Shaffer ML Kiely DK Mitchell SL . Survival and comfort after treatment of pneumonia in advanced dementia. Arch Intern Med. 2010;170(13):1102-1107. doi:10.1001/archinternmed.2010.181. 20625013 PMC2914628

[32] Fabiszewski KJ Volicer B Volicer L . Effect of antibiotic treatment on outcome of fevers in institutionalized Alzheimer patients. JAMA. 1990;263(23):3168-3172. 1693407

[33] van der Steen JT Lane P Kowall NW Knol DL Volicer L . Antibiotics and mortality in patients with lower respiratory infection and advanced dementia. J Am Med Dir Assoc. 2012;13(2):156-161. doi:10.1016/j.jamda.2010.07.001. 21450193 PMC6290468

[34] D’Agata E Loeb MB Mitchell SL . Challenges in assessing nursing home residents with advanced dementia for suspected urinary tract infections. J Am Geriatr Soc. 2013;61(1):62-66. doi:10.1111/jgs.12070. 23311553 PMC3545416

[35] Clark MD Halford Z Herndon C Middendorf E . Evaluation of antibiotic initiation tools in end-of-life care. Am J Hosp Palliat Care. 2022;39(3):274-281. doi:10.1177/10499091211027806. 34169763

[36] Dagli O Tasdemir E Ulutasdemir N . Palliative care infections and antibiotic cost: a vicious circle. Aging Male. 2020;23(2):98-105. doi:10.1080/13685538.2019.1575353. 30821574

[37] Juthani-Mehta M Malani PN Mitchell SL . Antimicrobials at the end of life: an opportunity to improve palliative care and infection management. JAMA. 2015;314(19):2017-2018. doi:10.1001/jama.2015.13080. 26426830 PMC4675049

[38] Levin PD Simor AE Moses AE Sprung CL . End-of-life treatment and bacterial antibiotic resistance: a potential association. Chest. 2010;138(3):588-594. doi:10.1378/chest.09-2757. 20472860

[39] Barlam TF Cosgrove SE Abbo LM , et al. Implementing an antibiotic stewardship program: guidelines by the infectious diseases society of America and the society for healthcare epidemiology of America. Clin Infect Dis. 2016;62(10):e51-e77. doi:10.1093/cid/ciw118. 27080992 PMC5006285

[40] Datta R Topal J McManus D , et al. Perspectives on antimicrobial use at the end of life among antibiotic stewardship programs: a survey of the society for healthcare epidemiology of America research network. Infect Control Hosp Epidemiol. 2019;40(9):1074-1076. doi:10.1017/ice.2019.194. 31328703 PMC7165360

[41] Quill T Miller F . Palliative Care and Ethics. Oxford, UK: Oxford University Press, Incorporated; 2014.

[42] Gordon M . Ethical challenges in end-of-life therapies in the elderly. Drugs Aging. 2002;19(5):321-329. doi:10.2165/00002512-200219050-00001. 12093319

[43] Kates OS Krantz EM Lee J , et al. Association of physician orders for life-sustaining treatment with inpatient antimicrobial use at end of life in patients with cancer. Open Forum Infect Dis. 2021;8(8):ofab361. doi:10.1093/ofid/ofab361. 34395710 PMC8360239

[44] Di Giulio P Toscani F Villani D Brunelli C Gentile S Spadin P . Dying with advanced dementia in long-term care geriatric institutions: a retrospective study. J Palliat Med. 2008;11(7):1023-1028. doi:10.1089/jpm.2008.0020. 18788965

